# Extreme Oncoplastic Breast Surgery: A case series of three patients from a lower-middle income country

**DOI:** 10.1016/j.amsu.2022.104899

**Published:** 2022-11-17

**Authors:** Narmeen Giacaman, Diala Abu Al-Halawa, Salem M. Tos, Mohammad G. Ibdah, Khaled Sharaf

**Affiliations:** a Faculty of Medicine, Al-Quds University, Jerusalem, Palestine; b Consultant Oncoplastic Breast Surgeon, Augusta Victoria Hospital, East Jerusalem, Palestine

**Keywords:** Oncoplastic breast surgery, Breast cancer, Good outcome, Case series

## Abstract

**Introduction:**

Oncoplastic breast surgery has become a major player in modern breast surgery.

It broadens the indications for breast-conserving surgery. More challenging cases are being treated more with what so-called “extreme oncoplastic breast surgery” which is defined as a breast-conserving operation, using oncoplastic techniques, in a patient who, in most physicians' opinions, requires a mastectomy.

**Methods:**

Replacement and/or displacement oncoplastic techniques with contralateral symmetrization to three female patients with breast cancer were done by an oncoplastic breast surgeon.

**Outcomes:**

The three patients had smooth recoveries with good aesthetic,oncologic and psychological outcomes.

**Conclusion:**

Oncoplastic breast surgery can be a better option than mastectomy with good oncologic, Psychological and aesthetic outcomes, even with extreme cases, yet long-term studies are needed.

## Highlights

• EOBS is defined as a breast conserving operation in a patient who, in most physicians' opinions, requires a mastectomy.
• The so-called EOBS is being used more frequently to treat challenging breast cancer cases.
• Oncoplastic breast surgery can be a better option than mastectomy with good aesthetic, oncologic and psychological outcome.

## 1 Introduction

Breast-conserving surgery has become the gold standard surgical care for early breast cancers with no survival difference compared to mastectomy [1]. Oncoplastic breast surgery combines the concept of breast-conserving surgery and optimum aesthetic outcome. It includes using plastic surgery techniques to reshape the breast after wide local excision. Many oncoplastic approaches have been proposed during the last decades but few cases have been described for multifocal or multicentric cancers [[2], [3], [4], [5], [6], [7], [8]].

Since achieving a negative margin is the major requirement for wide local excision, the so-called “extreme oncoplastic breast surgery” has emerged which is defined as a breast-conserving operation, using oncoplastic techniques, in a patient who, in most physicians' opinions, requires a mastectomy [9,10]. It allows successful breast conservation in large or multicentric tumors. It usually combines more than one technique of oncoplastic surgery. These procedures might provide a better quality of life than mastectomy, reconstruction, and radiation therapy [9]. Moreover, a good cosmetic result following breast conservation allows the patient to live her life with almost completely preserved sensitivity [10].

We present to you 3 cases in which extreme application of oncoplastic breast surgery was applied with good oncologic and aesthetic outcomes.

This case series has been reported in line with the PROCESS Guideline [11].

## 2 Case presentation

### 2.1 Case 1

**Fig. 1:**
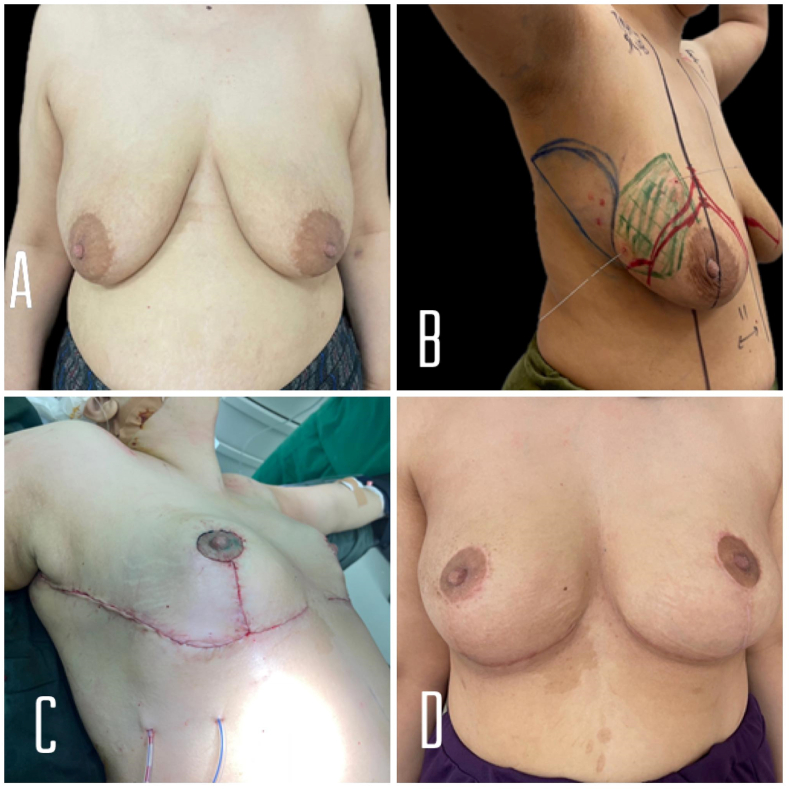
A: preoperative anterior view of both breasts. B: her markings and wires just before surgery (the green zone corresponds to her estimated tumor size). C:immediate result on the operating table **D**: 3 months after finishing radiotherapy with soft breasts and overall good aesthetic outcome. (For interpretation of the references to colour in this figure legend, the reader is referred to the Web version of this article.)

A 36-year-old lady with a free family and past medical history has presented with a right breast lump. A clinical exam showed tissue thickening in the lateral aspect of the right breast (Fig. 1 A and B).

The mammogram showed multiple suspicious lesions associated with microcalcifications in the Upper-outer quadrant (UOQ). Breast US showed few hypoechoic masses extending from 8–1 o'clock associated with enlarged ipsilateral axillary lymph nodes.

Core biopsy results showed intraductal carcinoma grade III, ER+, PR+, and HER2-. Genetic testing was negative for BRCA1, BRCA2, TP53, PALP2, CDH1and PTEN mutations. Staging CT scan imaging failed to reveal any distant metastasis.

She was started on neoadjuvant chemotherapy AC*4 + Taxol*12. Follow-up US showed a mild response.

The patient had undergone wire-guided (bracketed wires) therapeutic inverted “T” mastopexy, extended superomedial pedicle, and lateral intercostal artery perforator (LICAP) flap with sentinel lymph node biopsy converted to axillary dissection (after positive Lymph nodes on the frozen section) for the right side. Left inverted “T” Mastopexy, central mound, was performed on the left breast for symmetrization.

Wide local excision was performed with additional superior, medial and inferior cavity shaves (no more lateral tissue was left), and the total weight excised was 200 g. Initial reconstruction was made with lateral Hemi-rotation, then a lateral intercostal artery perforator flap was performed based on 3 perforators (Fig. 1. C).

Post-operative histopathology revealed multifocal residual moderately differentiated invasive ductal mammary carcinoma. Some scattered foci of high-grade comedo-type DCIS are seen. All the surgical resection margins, together with superior, inferior, and medial margins extensions are free of tumors. Five out of twenty examined lymph nodes are found metastatic (5+/20). And the Tumor stage was ypT1cN2aMX, multifocal post-chemotherapy. She had a smooth recovery followed by adjuvant radiotherapy and hormonal therapy.

She had a smooth recovery followed by adjuvant radiotherapy and hormonal therapy. She is shown 3 months after finishing radiotherapy with soft breasts and overall good aesthetic outcome (Fig. 1.D).

### 2.2 Case 2

**Fig. 2:**
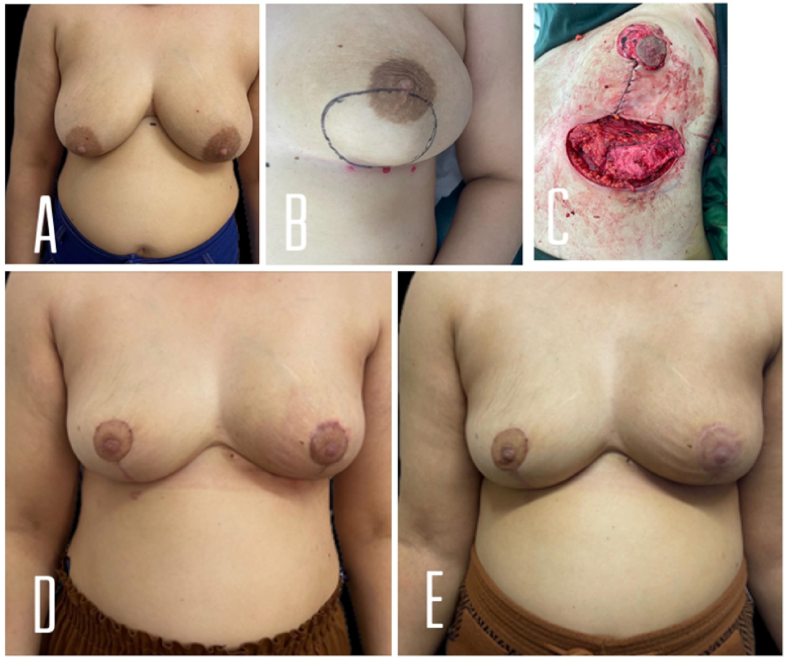
A and B: The patient's breasts pre-operatively and the estimated extent of the tumor in the left breast. C: intraoperative view of the left breast. D: postoperative 7 weeks picture of breasts E: 1 month post adjuvant radiotherapy.

A 33-year-old lady with a free past medical history and negative family history of malignancy has presented with a left breast and axillary lumps for 2 months. Clinically, there was a big lump occupying most of the lower pole (Fig. 2. A and B).

Breast US was performed and showed an ill-defined mass in the lower part at 5–6 o'clock, with suspicious micro-calcifications measuring 4.5 cm, located about 3 cm from the nipple, as well as a couple of well-defined hypoechoic lesions, the largest 0.5 cm, multiple pathological LNs, the largest 3.5 cm.

Core biopsy showed invasive ductal carcinoma, grade III, triple-negative breast tumor with Ki-67 40%. Staging CT showed no distant metastasis. She was started on neoadjuvant chemotherapy TAC x 6. On follow-up post neoadjuvant therapy, the mammogram was performed and showed a 7 cm mass in the lower pole of the left breast.

Left therapeutic mastopexy, superior pedicle, and Anterior Intercostal Artery Perforator (AICAP) flap with left axillary dissection were performed. Also, Right vertical mastopexy with a short horizontal scar was performed for symmetrization. The lumpectomy weight was 325 g anterior intercostal artery perforator flap was raised based on 2 perforators and tucked under the pillars of mastopexy. Fig. 2. C shows an intra-op view with an AICAP flap (Fig. 2. C).

The histopathological exam showed Minimal residual of poorly differentiated invasive ductal carcinoma in a background of chronic inflammation, all margins were free of malignancy. Metastasis in 2 lymph nodes of 33 examined lymph nodes of the first level of axillary lymph nodes, No metastasis in 12 examined lymph nodes of level 2 lymph nodes. And the Tumor stage was ypT1N1Mx.

She had a smooth recovery with a pleasant aesthetic result. She is shown at 7 weeks post-op and 1-month post adjuvant radiotherapy (Fig. 2.D and E).

### 2.3 Case 3

**Fig. 3:**
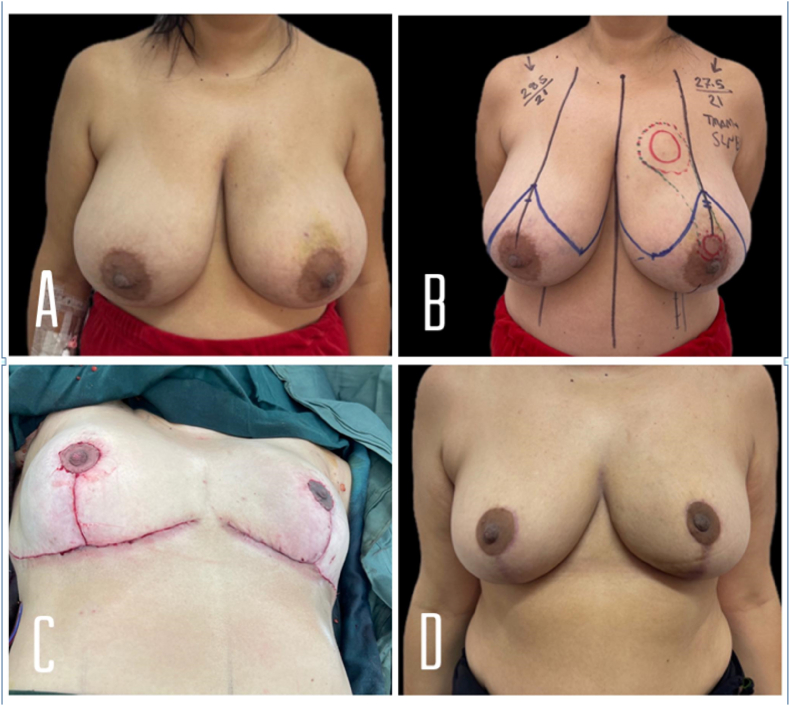
**A:** shows her breasts pre-operatively. **B:** immediate pre-op picture with the two tumors marked with red circles, the two tumors and intervening tissue was removed en bloc. **C:** immediate post-op result on surgical table. **D**: 3 months post-op. (For interpretation of the references to colour in this figure legend, the reader is referred to the Web version of this article.)

A 42-year-old lady with a free past medical history and negative family history of malignancy presented with a left breast lump (Fig. 3. A). US imaging showed an irregular lump at the 10th o'clock of the left breast 14 cm from the nipple and 1.3 cm from the skin measuring 3 × 2 cm BIRADS 6. A similar lesion located in the retroareolar region is also present measuring 1.5 × 1 cm. BIRADS 4, no axillary lymph node enlargement.

Core biopsy from the superior lump revealed an Intraductal carcinoma III, ER+, PR+, HER2-, Ki-67 25%. Core biopsy from the retroareolar lesion showed intraductal carcinoma III, ER+, PR+, HER2-, Ki-67 30%. Staging CT scan imaging showed no distant metastasis.

She was offered a mastectomy elsewhere but she refused due to cosmetic reasons.

Her Oncoplastic Breast surgeon decided to perform Left therapeutic mammoplasty (wise pattern, extended lateral pedicle technique) with sentinel lymph node biopsy and right reduction mammoplasty for symmetrization. The two tumors present high in UIQ and in the retroareolar lesion were removed en bloc (lumpectomy weight = 250 g) (Fig. 3. B). additional tissue was removed to reshape the breast with the total weight excised = 684 g. On the right side, a wise pattern of superomedial pedicle-based breast reduction was utilized. Fig. 3. C shows her immediate result on the operating table (Fig. 3. C).

The pathology report for the removed tissue showed multifocal poorly differentiated invasive ductal carcinoma, grade III, The greatest dimension of the largest tumor measured 3.3 cm. All margins are free of malignancy. 4 lymph nodes are free of malignancy. And the Tumor stage was pT2pNO. She started on adjuvant chemotherapy which was well tolerated.

Post-op she had mild wound dehiscence at the T junction of her left breast that was treated conservatively. Fig. 3.D shows her breast picture 3 months post-op (Fig. 3.D).

## 3 Discussion

The current case series discusses the use of oncoplastic breast-conserving surgery (OPBS) in the treatment of multifocal (MF) and multicentric (MS) breast cancer. The treatment of breast cancer has been widely explored in an attempt to achieve better oncological and cosmetic outcomes. Yet, there's still a need to explore the applicability of various novel surgical techniques in large-sized breast cancers, especially in low- and middle-income countries [12,13].

It has become evident that breast-conserving surgeries bear no increased oncological risk in terms of local recurrence when compared with mastectomy [14], and even higher overall survival rates [15,16]. Breast-conserving surgeries have been increasingly becoming the surgical approach for MF and MS breast cancers. Moreover, the addition of OPBS to breast-conserving techniques has been gaining wide acceptance [17,18].

In cases requiring large excisions, the oncoplastic breast surgery (OPBS) techniques represent a state-of-the art way to avoid major deformities of the breast [19].

As demonstrated in this case series, OPBS provides great cosmetic results, which adds to the literature that has found OPBS leading to better cosmetic results when compared with standard conserving approaches [20], OPBS can potentially expand the inclusion criteria of women applicable for breast-conserving techniques, where tumor to breast size ratio is high. Studies have demonstrated that OPBS combined with breast reduction can provide larger surgical margins, therefore enhancing cosmetic outcomes [21].

In terms of the oncological outcome of OPBS, many have discussed its impact on local and overall recurrence of breast cancer. In a meta-analysis of 11 studies, Chen et al. concluded that the addition of OPBS to breast-conserving techniques results in less re-excision rates and similar long-term survival rates when compared to breast-conserving surgeries alone [22]. Moreover, other studies have found that positive margins seem to be less frequent when applying OPBS techniques compared to breast-conserving surgery alone [23,24].

In regard to postoperative complications, it has been found that postoperative complications of OPBS using partial mastectomy offer a similar complication rate to traditional mastectomy [25]. Additionally, OPBS allows the use of patient tissue for breast reconstruction, thus avoiding the need for breast implants. This could provide less postoperative complications, where postoperative radiotherapy can cause complications when applied to breast implants, including contractures, leaks, and rupture [26].

In this case series, we aim not only to explore and present the benefits of OPBS in the treatment of breast cancer but also to highlight the importance of using these techniques in the Palestinian context. In Palestine, breast cancer constitutes 15% of cancer cases among women [27]. There is a need for enhancing the quality of care for breast cancer among women, and as demonstrated in this case series, OPBS techniques can provide a considerable advancement in the quality care among Palestinian women, thereby enhancing both the health and the psychological wellbeing of breast cancer survivors.

## 4 Conclusion

The so-called “extreme oncoplastic surgery” is emerging as a new promising concept in breast cancer surgery and can be a better option than mastectomy with good oncologic and aesthetic outcomes, in selected patients with large and/or multicentric tumors, yet long-term studies are needed.

## Human and animal rights

No animals were used in this research. All human research procedures followed were in accordance with the ethical standards of the committee responsible for human experimentation (institutional and national), and with the Helsinki Declaration of 1975, as revised in 2013.

## Ethical approval

Informed consent was signed by all three patients.

## Sources of funding

The author has no source of funding

## Provenance and peer review

Not commissioned, externally peer reviewed.

## Authors’ contributions

Study concept or design: Khaled Sharaf, Narmeen Giacaman.

Writing the manuscript: Narmeen Giacaman, Diala Abu Al-Halawa, Salem M. Tos, Mohammad G. Ibdah, Khaled Sharaf.

Review & editing the manuscript: Narmeen Giacaman, Khaled Sharaf.

## Declaration of competing interest

The authors declare no conflicts of interest.

## Abbreviations

AC: Adriamycin and cyclophosphamide.
BRCA1: BReast CAncer gene 1
BRCA2: BReast CAncer gene 2
TP53: tumor protein 53
PALB2: partner and localizer of the BRCA2 gene
PTEN: Phosphatase and TENsin homolog deleted on chromosome 10
ER: Estrogen receptor
PR: Progesterone receptor
HER-2: human epidermal growth factor receptor 2
BIRADS: Breast Imaging Reporting and Data System
